# Tactile-to-Visual Cross-Modal Transfer of Texture Categorisation Following Training: An fMRI Study

**DOI:** 10.3389/fnint.2018.00024

**Published:** 2018-06-07

**Authors:** Georgia O’Callaghan, Alan O’Dowd, Cristina Simões-Franklin, John Stapleton, Fiona N. Newell

**Affiliations:** Trinity College Institute of Neuroscience, School of Psychology, Trinity College Dublin, Dublin, Ireland

**Keywords:** crossmodal, multisensory, vision, touch, texture perception, material perception, categorisation, practise

## Abstract

We investigated the neural underpinnings of texture categorisation using exemplars that were previously learned either within modalities (visual training and visual test) or across modalities (tactile training and visual test). Previous models of learning suggest a decrease in activation in brain regions that are typically involved in cognitive control during task acquisition, but a concomitant increase in activation in brain regions associated with the representation of the acquired information. In our study, participants were required to learn to categorise fabrics of different textures as either natural or synthetic. Training occurred over several sessions, with each fabric presented either visually or through touch to a participant. Pre- and post-training tests, in which participants categorised visual images only of the fabrics, were conducted during a functional magnetic resonance imaging (fMRI) scan. Consistent with previous research on cognitive processes involved in task acquisition, we found that categorisation training was associated with a decrease in activation in brain regions associated with cognitive systems involved in learning, including the superior parietal cortex, dorsal anterior cingulate cortex (dACC), and the right dorsolateral prefrontal cortex (DLFC). Moreover, these decreases were independent of training modality. In contrast, we found greater activation to visual textures in a region within the left medial occipital cortex (MOC) following training. There was no overall evidence of an effect of training modality in the main analyses, with texture-specific regional changes associated with both within- (visual) and cross- (touch) modal training. However, further analyses suggested that, unlike categorisation performance following within-modal training, crossmodal training was associated with bilateral activation of the MOC. Our results support previous evidence for a multisensory representation of texture within early visual regions of the cortex and provide insight into how multisensory categories are formed in the brain.

## Introduction

The issue of how sensory information is organised into different categories has received considerable interest in the literature, particularly with regards to object shapes (Riesenhuber and Poggio, 1999; Newell and Bülthoff, 2002), faces and facial expressions (Calder et al., 1996; Bülthoff and Newell, 2004) and scenes (Thorpe et al., 1996; Greene et al., 2016). In contrast, the question of how multiple sensory inputs contribute to the formation of categories has received relatively little attention. In principle, perceptual categories should be formed using all relevant information about a concept (e.g., Taylor et al., 2006). For example, a Labrador belongs to the category of ‘dog’ on the basis of multisensory information including visual information about its shape and size, as well as other non-visual information such as the sound of the dog barking, the feel of its coat and maybe even its smell. Yet despite this sensory convergence, the cognitive and cortical processes underpinning the formation of multisensory categories is poorly understood.

A number of cognitive processes are known to be involved in the formation of categories based on novel exemplars. In particular, the task of categorisation itself involves learning to associate particular features of an object with belonging to a particular category. Such a skill is likely to be domain general, and non-specific to different category types. The acquisition of this skill can be rapid, if the features associated with each category are distinctive, or more effortful if either the distinguishing features are subtle (e.g., Biederman and Shiffrar, 1987) or the exemplars from each category share common features (Newell and Bülthoff, 2002). In the latter case, practise on the task is likely to lead to better categorisation performance. Concomitant with the cognitive ability to form categories is an increase in familiarity with the properties of the particular exemplars themselves during task acquisition. Thus, the ability to categorise objects becomes more efficient with increasing familiarity of exemplars within each object category as well as perceptual expertise in distinguishing between these exemplars (see e.g., Bornstein and Mash, 2010). However, what is not clear is how exemplar familiarity or expertise in one sensory domain transfers to another modality. In the following study, we used neuroimaging to investigate the role of higher-level cognitive and lower-level perceptual processes involved in learning to categorise novel textures and ask whether these processes may be specific to, or independent of, the learning modality.

The capacity to learn and become proficient in a new task is present throughout the lifespan, with practise serving as an important cornerstone of such learning (Ericsson et al., 1993; Ericsson and Charness, 1994; Jonides, 2004). Indeed, there is evidence that repeated practise not only influences behavioural efficiency, by increasing speed and/or accuracy (Chein and Schneider, 2005), but also by altering the functional and/or structural properties of the brain (Kelly and Garavan, 2004; Chein and Schneider, 2005, 2012). Task acquisition can influence brain activity through various individual mechanisms, including a reorganisation or redistribution of neural activity within and across neural networks which can manifest as increases or decreases in brain activity during the period of training (Petersen et al., 1998; Poldrack, 2000; Kelly and Garavan, 2004). More specifically, reorganisation refers to fundamental changes in the neural systems underlying task acquisition, such that neural activity may subside in one region and increase in another depending on the cognitive strategies adopted (e.g., see Petersson et al., 1999). Conversely, redistribution refers to the reduced involvement of neural systems involved in attention and cognitive control (the so-called ‘scaffolding’ network; Kelly and Garavan, 2004) and an increase in activity in regions underlying the sensory representation of the task stimuli (e.g., see Frutiger et al., 2000).

There is clear evidence that training in a particular task can exert significant changes on brain function (Poldrack, 2000; Kelly and Garavan, 2004; Carmel and Carrasco, 2008; Sasaki et al., 2010). However, reports of functional changes to task- and stimulus-specific regions are somewhat heterogeneous, with patterns of functional activity associated with either increases or decreases in strength following training in both higher cognitive regions as well as lower, sensory nexuses. Moreover, these effects can vary across different classes of stimuli, stimulus familiarity, characteristics of the training procedure and training groups (Schwartz et al., 2002; Carmel and Carrasco, 2008; Sasaki et al., 2010). Other studies have provided evidence to suggest that plasticity can occur within the primary visual cortex in a stimulus-driven manner, that is, independently of top-down modulation (Karni and Sagi, 1991; Schwartz et al., 2002; Pourtois et al., 2008). For example, following visual texture discrimination training, Schwartz et al. (2002) observed an increase in functional activity in the visual cortex that appeared to be unrelated to functional changes in other brain regions. A similar finding was demonstrated in an fMRI study by Yotsumoto et al. (2008) in which participants were also trained to visually discriminate target textures located in specific quadrants of the visual field. In an attempt to unify these findings, Chein and Schneider (2012) proposed a Triarchic Theory of Learning in which they outlined predictions for the involvement of different brain areas during different stages of task acquisition. In summary, they proposed that both task acquisition and proficiency are associated with a decrease in activation in regions of the brain involved in metacognition or cognitive control as well as an increase in regions of the brain that are more involved with the representation of the stimulus-specific information required for the task.

Texture information can be perceived using vision or touch, but often in combination. However, it is not clear whether learning in each of these modalities transfers to the other for texture perception. Although some studies on texture perception have provided evidence for a multisensory representation, other behavioural and neuroimaging findings suggest that vision and touch may contribute in different ways to the perception of texture. For example, Podrebarac et al. (2014) reported texture- but not shape- selective processing in regions of the occipitotemporal cortex but found no evidence for overlapping activation to visual or tactile inputs across these regions. Furthermore, in their review of behavioural and neuroimaging studies on crossmodal texture perception, Whitaker et al. (2008) argued for the independent processing of texture across vision and touch. They acknowledged, however, that studies of texture often involved distinct aspects relating to either the spatial distribution of texture components (such as raised dots) or the roughness of surface textures and that further research was required to investigate crossmodal interactions for the purpose of perceiving more naturalistic textures.

In contrast, other studies have provided evidence for overlapping regions within the visual and parietal cortices that are selective to texture processing within both the visual and tactile modalities. For example, Stilla and Sathian (2008) reported that a haptic texture matching task activated a number of regions that were selective for texture but not for shape, including ventral somatosensory areas, the parietal operculum and bilateral posterior insula. These regions were also identified as activated during the tactile exploration of textures (see e.g., Simões-Franklin et al., 2011). Furthermore, Stilla and Sathian, along with other studies, reported activation to haptic texture perception in areas of the visual cortex that also overlap with regions activated to visual textures, specifically the middle occipital cortical region extending to the middle occipital gyrus (see also Sathian et al., 2011; Sathian, 2016). Most relevant to the goal of our study are findings suggesting a distribution in activations from low-level, image-based analyses of texture within early regions of sensory cortices (visual or touch) to more perceptual regions within the brain. For example, Hiramatsu et al. (2011) reported a distributed activation pattern to visual textures that included the primary visual cortex and higher-level areas such as the collateral sulcus (see e.g., Cant and Goodale, 2011). Similarly, Sathian et al. (2011) found distributed activations to tactile textures from somatosensory regions to the more higher-level medial occipital cortex (MOC). Using a more direct comparison of regions activated to visual and tactile textures, Eck et al. (2013) compared the associated BOLD response during a texture matching task across bimodal and unimodal exploration (most notably for the purpose of our study, participants were not required to conduct a cognitive task). Consistent with previous studies, Eck et al. (2013) found distributed activation across a network of cortical regions including early sensory areas (e.g., the post-central gyrus activated by haptic exploration) to higher-order areas involved in perception such as the middle occipital gyrus, collateral sulcus and lingual gyrus (see Sathian, 2016 for a review).

The current study sought to examine how a short-term training programme influences the functional characteristics of brain networks. Specifically, we included a training paradigm to elucidate the neural substrates underpinning the crossmodal transfer of learned information during texture categorisation, as this is currently poorly understood. Participants learned to categorise textures as either ‘natural’ or ‘synthetic’ through either touch or vision and were subsequently tested on their ability to categorise these trained textures using vision only. Consistent with Chein and Schneider’s (2012) Triarchic model of Learning, we hypothesised that decreases in the cognitive ‘scaffolding’ network, including metacognitive systems, would be observed as participants became more proficient at the texture categorisation task itself (see also Kelly and Garavan, 2004). Furthermore, we hypothesised that brain regions associated with the perceptual representation of texture would become more activated with practise. In light of previous studies on texture perception across modalities, we expected an increase in activation in cortical regions associated with texture perception and that those regions, if multisensory, would be equally activated by unimodal and crossmodal texture information.

## Materials and Methods

### Participants

Seventeen volunteers (9 male) were recruited from the undergraduate and postgraduate student population at Trinity College Dublin via local advertising. All reported to be right-hand dominant (verified through the Edinburgh Handedness Inventory) and had normal or corrected-to-normal vision. All reported to be healthy and all confirmed no history of neurological, psychiatric or psychological illness. One of the participant’s data set was excluded from the final analysis as they failed to show any effect of training and did not complete the study. The final sample included eight males and eight females, with a mean age of 21.2 (1.68) years (range from 19 to 24). The experimental protocol was approved by the Psychology Research Ethics Committee at Trinity College Dublin prior to testing. Informed, written consent was obtained prior to study participation and compensation was at a rate of €5.00 per hour or in exchange for undergraduate ‘research credits’ to complete course requirements.

### Stimuli and Apparatus

**FIGURE 1:**
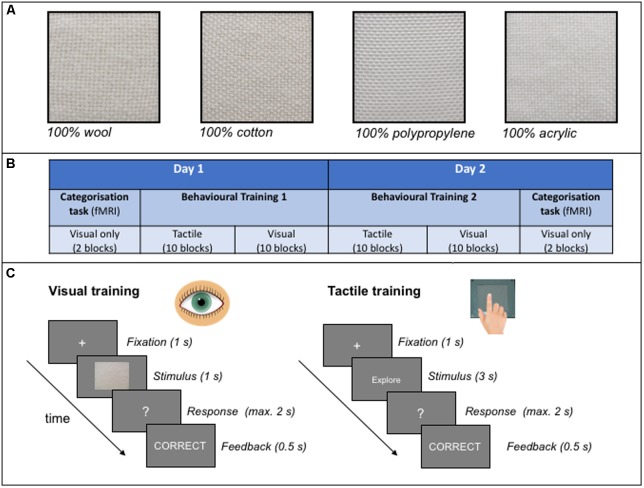
**(A)** An example of some of the pure (100%) fabrics from either the natural (wool, cotton) or synthetic (polypropylene, acrylic) categories used as stimuli in the study. All samples shown are examples of coarse textures. **(B)** A schematic illustration of the experimental protocol adopted in the study. **(C)** An example of a typical trial structure in the visual and tactile behavioural training sessions. See text for further details.

The stimuli were comprised of a range 32 different samples of texture fabrics. Each sample varied in weave (coarse or fine textures) and the quantity of ‘natural’ fabric contained in the threads. The coarse and fine textures were defined on the basis of the spatial period of the weave, with fabrics of less than, or greater than, 0.2 mm spatial period defined as ‘fine’ and ‘coarse,’ respectively (Hollins and Bensmaïa, 2007). The addition of both fine and coarse types ensured we had a representative sample of roughness textures in our study, but this dimension was not of relevance to the overall hypothesis of the study. The ‘natural’ fabrics included cotton and wool, whereas the synthetic fabrics included polypropylene and acrylic. All fabric samples were custom woven as samples of pure ‘natural’ (i.e., 100% cotton or 100% wool) or pure synthetic (i.e., 100% polypropylene or 100% acrylic) fibres. Other samples included mixtures of two fibres, one each from the natural and synthetic category. All mixtures contained either 75% of one fibre and 25% of the other fibre category, or 50% of each, and fibres were evenly distributed within each sample material. For example, a material sample may contain 75% wool threads and 25% acrylic threads, or vice versa. **Figure 1A** provides an illustration of some of the pure fabric samples used as stimuli in this study. Prior to conducting the study, we determined that the correct categorisation of samples containing 75% or more of a natural fibre was ‘natural’ (100% wool, 100% cotton, 75% wool and 75% cotton). For the samples that contained 50% natural fibres, we determined that the correct categorisation of 50% wool was ‘natural’ and 50% cotton was ‘synthetic.’ This was based on the results of a previous pilot study where the 50% wool mixes were consistently perceived as being ‘natural’ whereas the 50% cotton mixes were consistently categorised as ‘synthetic’^1^. Finally, all the remaining samples were categorised as synthetic (25% wool, 25% cotton, 0% wool and 0% cotton).

Each of the fabric samples were mounted onto a hard plastic background surface and secured in place by a frame. The edges of the fabric were fixed behind the block so that these could not be viewed or felt during exploration. The dimensions of each fabric sample was 80 mm in length and 80 mm in width. Each fabric sample was photographed under standardised lighting conditions that simulated diffuse, normal daylight. For both the visual training and fMRI categorisation testing sessions, high-resolution images of the samples were presented on a computer monitor during the training task, or projected onto a screen located at the bore of the MRI system during the fMRI task. The same visual images of the 32 fabrics were used for both the (fMRI) categorisation and behavioural training sessions.

During the behavioural training sessions only, the apparatus consisted of a table at which the participant sat and a curtain which prevented the participant from viewing the stimuli during tactile exploration. The participant and experimenter sat on either side of the curtain. For tactile presentation of the stimuli, the fabrics were placed behind a curtain, the participant reached underneath the curtain and the experimenter manually presented the stimuli one at a time.

All tasks (behavioural and fMRI) were programmemed and presented using Neurobehavioural Systems (NBS) Presentation software (version 0.70^2^).

### Design

The main experimental protocol was based on an ABA design in which the effects of a training intervention (B) was measured by comparing texture categorisation performance pre- and post-training (A) (see **Figure 1B** for a schematic illustration of the protocol). Performance was measured as behavioural responses (accuracy) as well as functional changes in the brain (BOLD response) as a consequence of training. Participants were presented with all fabric samples throughout the experiment.

During the behavioural training session, participants were trained over 10 blocks of trials within each training modality per day, over 2 days (as illustrated in **Figure 1B**). Thus there were 20 training blocks in total in each of two main training sessions. Participants were offered a self-timed break between blocks. Each block included 32 samples presented once in either the tactile or visual modality: each participant learned to categorise half of the samples (16) using touch only, and the other half using vision only. Participants were randomly allocated to the training modality condition. Fabric texture at training (coarse or fine) was also counterbalanced across participants such that half of the participants were trained on fine textured fabrics in one modality and coarse fabrics in the other modality.

The pre- and post- training categorisation task was always conducted in the visual domain only and fMRI was simultaneously acquired during these sessions. During the categorisation task, responses (‘natural’ or ‘synthetic’) were indicated using the index and middle fingers of the participant’s right hand with a 1 × 2 Fibre-Optic response pad. The button assigned to ‘natural’ (either middle or index finger) was counterbalanced across participants. Within each categorisation block, all 32 images of texture stimuli were shown twice (once in each of two blocks) and were presented in a randomised order across participants, irrespective of the training modality to which each participant was allocated.

### Procedure

The experiment took place over two consecutive days (see **Figure 1B**). There were two main sessions to this study: an intensive behavioural training session and a visual categorisation task which was conducted twice, both before and after training. Participants completed each of the visual categorisation tasks in conjunction with fMRI on the 1st and 2nd day. On day 1, the first visual categorisation task in the scanner was followed by 2 hours of behavioural training (i.e., outside the scanner). On the 2nd day participants undertook two further hours of behavioural training before conducting a final visual categorisation task in the scanner.

#### Behavioural Training

During this session, participants learned to categorise each fabric sample as either ‘natural’ or ‘synthetic’ through feedback. As shown in **Figure 1C**, a trial started with a fixation cross of 1 s which prepared the participant for the subsequent presentation of each stimulus. The structure of the trials was similar across modalities with the following exceptions: each image of a fabric was presented to the participant for 1000 ms in the visual modality and 3000 ms in the tactile modality. For tactile training, each sample was presented behind a curtain and the participant was instructed to explore the sample using circular hand movements in an anti-clockwise direction (at an average rate of one circular movement per second). An inter-trial interval of approximately 3 s occurred in the tactile modality only to allow the experimenter to change the fabric stimuli between trials. Participants verbally reported their responses and feedback was provided to the participant immediately after a response was made to each trial.

#### Categorisation Task (fMRI Acquisition)

The pre- and post- training categorisation task was conducted in the visual modality only. As with the training study, participants were required to categorise each visual image as either ‘natural’ or ‘synthetic.’ Each test session was conducted in the visual modality alone, thus the related test session was either with stimuli presented within the same modality or across modalities to the training modality.

Participants completed four blocks of each test whilst being scanned using fMRI, two before and two after the behavioural training blocks. Each block lasted 300 s (150 TRs) and contained all 32 unique fabric samples, one per trial (with 64 samples across the two blocks in each categorisation task). A trial began with a fixation cross with a duration that was jittered between 4 and 10 s. The stimulus image was then presented for 1000 ms, followed by a response window of 2 s (question mark). No feedback was provided during the categorisation task.

### Magnetic Resonance Imaging (fMRI) Image Acquisition

The current study utilised a Philips Achieva 3.0 Tesla MR system in conjunction with an 8-channel head coil. A mounted mirror reflected a display that was projected onto a panel behind the participant’s head outside the magnet. After an initial reference scan to allow for the resolution of sensitivity variations, 180 high-resolution T1-weighted anatomic MPRAGE transverse images (FOV 230 mm, thickness 1.5 mm, voxel size 1.5 mm × 1.5 mm × 1.5 mm, total duration 343 s) were acquired for each participant, which allowed for subsequent activation localisation and spatial normalisation. Functional images consisted of 40 non-contiguous (0.3 mm gap), 3 mm transverse slices covering the entire brain and collected in ascending order. Images were acquired using a T2^∗^ weighted echo-planar imaging sequences (TR = 2000 ms, TE = 25 ms, FOV 240 mm, 80 × 80 matrix size in Fourier space). All imaging utilised a parallel sensitivity encoding (SENSE) approach with a reduction factor of 2.5 (Pruessmann et al., 1999).

### fMRI Preprocessing and Statistical Analysis

The analysis of fMRI data was conducted in Matlab 2016a (Matlab: MATLAB and Statistics Toolbox Release, 2016a) using Statistical Parametric Mapping, version 12 (SPM12, 2014). The imaging data initially underwent realignment, spatial normalisation to MNI space and smoothing with a Gaussian kernel of full-width half maximum (FWHM) 8 mm^3^. Volumes with scan-to-scan motion in excess of 1 mm were identified by the ArtRepair toolbox (by Mazaika et al., 2009) and were flagged to be deweighted in the design matrix phase. In the design matrices an additional motion regressor was included (i.e., a 7th motion regressor), with the onset and duration of these volumes marked. This occurred in less than 0.1% of scans.

General linear models (GLM) at the individual subject level were created for each of the four runs; two before training and two after training. The following events were modelled; tactile trained synthetic, tactile trained natural, visually trained synthetic, visually trained natural, with a canonical HRF and a high pass filter (HPF) of 128 Hz.

First level analysis consisted of contrasting the four conditions against baseline, separately for tests before and after training, leading to the creation of eight conditions; two training modalities (tactile or vision), categorisation task (pre- and post- training), and fabric category (synthetic or natural). Although fabric category was not directly related to our hypothesis, we included this factor for completeness to rule out any inherent differences in activation associated with natural or synthetic fabrics that may affect the overall results. Following this, the flexible factorial approach in SPM12 was used to apply a 2 × 2 × 2 within-subject ANOVA for categorisation test (pre- and post training), training modality (vision or tactile) and naturalness (synthetic vs. natural fabrics) for these first level contrasts at the group level. As there was no effect of the training schedule that participants were assigned to, the current model did not contain a between-groups factor to this effect.

Main effects and interactions from the second level analysis were reviewed with t-contrasts with an initial uncorrected voxel threshold of *p* < 0.001 and had to exceed a 0.05 Family wise error (FWE) rate corrected cluster extent of *k* > 104 (832 μl) continuous voxels, determined by AFNI’s 3dClustSim programme (Analysis of Functional NeuroImages, 2016) with the following settings; probability of a cluster *a* < 0.05; voxel threshold *p* < 0.001; autocorrelation function (ACF) values of 0.47, 4.52, and 10.68; one-tailed; edges touching.

Six further second level contrasts were produced as they addressed the particular hypotheses of the current study. Separate analyses of change in activation during the categorisation tasks pre- to post-training, and vice versa, were conducted for stimuli trained through vision (within modality) and through touch (cross modality). In addition, direct comparisons were made between activity in response to stimuli previously trained through touch and through vision, by contrasting these after training, i.e., ‘post- training vision > touch’ and ‘post-training touch > vision.’ Since these secondary analyses were exploratory, a more stringent threshold was applied with a cluster extent of *k* > 251 (2,008 μl), FWE corrected to 0.001, to account for multiple comparisons.

### Statistical Analyses of Behavioural Performance

Two-tailed *t*-tests on performance during each training session (visual and tactile) was used to assess improvement in trials presented at the end of the training session relative to those performed at the beginning. To assess behavioural changes in categorisation performance following training we used a 2 × 2 within-subjects ANOVA on participants’ mean accuracy scores with categorisation task (pre- or post- training) and test modality (within or across modalities) as factors. All analyses of behavioural data were conducted using the Statistical Package for Social Sciences (SPSS) version 24 (IBM Corporation; Armonk, NY, United States). Effect sizes are reported as partial eta-squared (η_p_^2^). Where appropriate, Tukey’s honest significant difference (HSD) pair-wise comparison tests, which corrected for multiple comparisons, were used to conduct *post hoc* analyses of any interactions and significant main effects from the ANOVA, where appropriate. All reported *p*-values were based on an alpha level of α = 0.05.

## Results

### Behavioural Performance

#### Behavioural Training Performance

We first measured performance to each block on the training task to ensure that participants were improving on the texture categorisation task during both visual and tactile training. Because some participants reported fatigue during the final training block (Block 20), and performance generally reached asymptote for all participants, we took the average performance between the final two training blocks and compared this to performance in the initial block (Block 1) of training trials, in each modality. Although the task was more difficult in the tactile modality (56.3 and 69.2% accuracy in Block 1 for touch and vision, respectively) there was evidence of improvement in both modalities: performance following training in the tactile modality improved by 7.5% (*SD* = 14.75%) whilst performance in the visual modality improved by 14.2% (*SD* = 6.45%). There was no difference in improvement of performance across the modalities [*t*(16) = 1.65, *p* = 0.11].

#### Categorisation Test Performance

**FIGURE 2:**
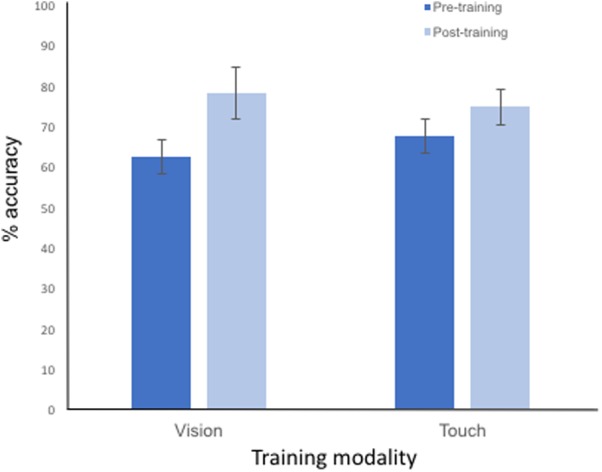
Plot showing the mean accuracy performance to each categorisation task conducted pre-training and post-training. The categorisation task was conducted using vision only and performance is shown to stimuli previously trained in the visual (i.e., within modality) and tactile (i.e., cross-modal) modalities. Error bars represent ±1 SEM.

A plot comparing the mean accuracy performance at test, (i.e., pre- and post- training), depending on visual or tactile training is presented in **Figure 2**. These pre- and post-training (categorisation) tests were conducted within the scanner, and in the visual modality only (therefore the cross-modal performance is indicated in the ‘Touch’ training condition). A 2 × 2 ANOVA, conducted on the participants’ accuracy at categorising the visual stimuli as either ‘natural’ or ‘synthetic’ identified a main effect of categorisation task [*F*(1,15) = 43.37, *p* < 0.001, η_p_^2^ = 0.756], with an improvement in accuracy performance in this task from pre- to post- training (as shown in **Figure 2**). No main effect of (within or across) training modality (*F* < 1) was observed. The interaction between test session and training modality approached but failed to reach significance [*F*(1,15) = 3.53, *p* = 0.079]. However, since this interaction was most pertinent to our predictions, a more detailed, *post-hoc* analyses of the categorisation task performance data confirmed significant pair-wise improvement in task performance from pre- to post-training sessions for each modality (Tukey HST; all *p*s < 0.05), and no difference in performance across the training modalities at either the pre-training or post-training tests (all *p*s > 0.1). These results are therefore consistent with the performance improvement during the behavioural training session itself, reported above.

### fMRI Results

**FIGURE 3:**
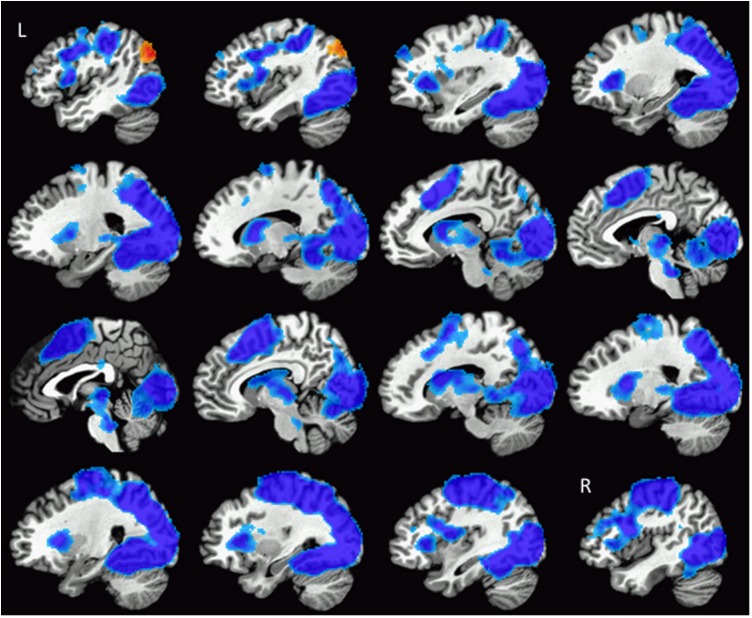
Illustration of the widespread pattern of changes in activation during texture categorisation following training. Regions showing reduced activity (blue colours) included bilateral aspects of the occipital cortex, IPS, primary and somatosensory cortices, SMA, dACC, DLPFC, insula, caudate, and putamen. The left MOC was the only region to show increased activation after training (red colours). Images are arranged such that the first slice at the top left of the figure is located in the left hemisphere (L), progressing rightward through the brain until the last slice in the right hemisphere (R) shown on the bottom right of the figure.

Global brain analysis identified a widespread pattern of regions that had significantly greater activation during the categorisation task before training compared to after training. Cortical regions within this bilateral, continuous cluster included the majority of the occipital cortex, the superior parietal cortex [including the intraparietal sulcus (IPS) and aspects of the primary motor and somatosensory cortices], supplementary motor area (SMA), dorsal anterior cingulate cortex (dACC), the dorsolateral prefrontal cortex (DLFC), putamen, caudate, and insula. Greater activation was also found in the thalamus prior to training. These regions are illustrated in **Figure 3**. This pattern of activation did not appear to be specific to the training modality: separate analyses of pre- to post-tactile and visual training showed decreased activation in these same regions.

**FIGURE 4:**
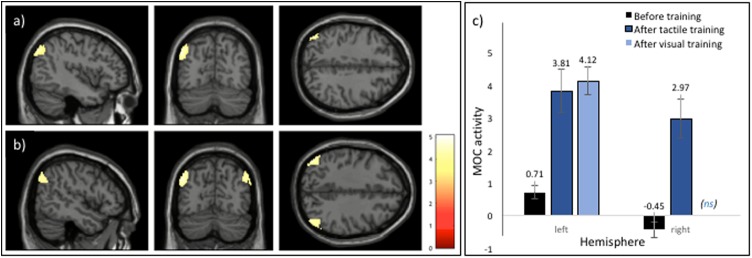
Regions demonstrating a significant increase in activation during the categorisation task post training. **(A)** A main effect of training was associated with increased activation in the left MOC. **(B)** An illustration of the result of subsequent analyses of the effect of training modality which revealed bilateral activation in the MOC following training using touch. **(C)** Barplot showing relative increases in both the left and right MOC during the visual categorisation task from before to after training, with after-training activity shown dependent on the training modality of vision or touch. Note that barplots depicting the mean peak activations post-training are shown only for regions associated with a significant increase in activation (i.e., activation in the right MOC to visual training was not significant, ns). See text for details on the cluster-based analyses conducted. Error bars represent ±1 SEM.

In addition, a region within the visual cortex, the left MOC demonstrated a significantly greater response after training (i.e., a main effect of training) as shown in **Figures 3**, **4A**. In a subsequent analysis, we separated the responses to the visual categorisation task by training modality (within or across), and found that this pattern remained for stimuli trained through vision (i.e., within modalities) but was bilateral for stimuli trained through touch (across modalities), as illustrated in **Figure 4B**. Values extracted from the left and right MOC^3^ associated with the visual and tactile categorisation task pre- to post training, are illustrated in **Figure 4C**. Finally, no main effects of naturalness (synthetic vs. natural) or training modality (vision vs. tactile training) were found, even with a direct *t*-test comparison between stimuli trained through vision and through touch during the post-training session only. All functional results are summarised in **Table 1**.

**Table 1:** Summary of the results of the whole brain analysis visual categorisation task (before vs. after training), training modality (within vs. across modality) and naturalness (synthetic vs. natural) as factors.

Side	Brain areas	Cluster extent (k)	Cluster volume (mm^3^)	Peak voxel T	Peak MNI coordinates (x, y, z)
**Main effects**					
*Before Training > After Training*					
R Middle Occipital Gyrus		60,868	486,944	11.07	36 -88 10
R Fusiform Cortex				10.82	30 -70 -4
R Fusiform Cortex				10.79	28 -52 -6
L Middle Frontal Gyrus (BA9/46)		213	1,704	4.74	-34 44 40
				3.29	-44 38 34
				3.21	-36 34 32
L Middle Frontal Gyrus (BA46)		109	872	4.48	-38 38 18
L Superior Frontal Gyrus (BA6)		135	1,080	3.9	-24 -10 58
*After Training > Before Training*					
L Middle occipital cortex		481	3,848	5.08	-42 -76 38
*Main effect of Training Modality (Vision or Touch)*					
No significant differences					
*Main effect of Naturalness (synthetic or natural fabric)*					
No significant differences					
*Training ^∗^ Training Modality Interaction*					
No significant differences					
**Further analyses**					
*Vision: Before > After Training*					
Same regions and approximate cluster sizes observed as for *‘Before Training > After training’*					
*Vision: After > Before Training*					
Same regions and approximate cluster sizes observed as for *‘After Training > Before Training’*					
*Touch: Before > After Training*					
Same regions and approximate cluster sizes observed as for *‘Before Training > After training’*					
*Touch: After > Before Training*					
Middle occipital (L)		598	4,784	5.07	-46 -76 38
Middle occipital (R)		328	2,624	4.22	48 -72 38
*After Training: Vision > Touch*					
No significant differences					
*After Training: Touch > Vision*					
No significant differences					

## Discussion

Our study was designed to elucidate the neural correlates of visual texture categorisation following both within and crossmodal (tactile) training. In particular, we were interested in determining the changes in functional activation that occurred following categorisation training of textures and whether the benefits of training in one modality transferred to another. Texture categorisation performance improved across participants following both visual and tactile training. Consistent with previous studies on practise effects, we found a decrease in activation with training in regions of the brain that are typically associated with higher cognitive processes (Kelly and Garavan, 2004; Chein and Schneider, 2012). These regions included areas within the parietal cortex, particularly the IPS, as well as regions within the anterior cingulate and prefrontal cortex. Furthermore, our findings also suggested an increase in activation in a region of the occipital cortex, the MOC, that has previously been associated with the crossmodal perception of textures (see e.g., Sathian, 2016). Thus, our findings are consistent with a redistribution of neural activity following learning on a texture categorisation task. More specifically, the results lend support to the Triarchic model of Learning proposed by Chein and Schneider (2012) in that an increase in proficiency at a texture categorisation task was associated with both a decrease in activation within the cognitive ‘scaffolding’ network, as well as an increase in activation within brain regions associated with the perceptual representation of texture.

Collectively, the activated regions within the prefrontal and parietal cortices represent a domain-general system and are associated with cognitive processing of information involved in the task, such as task acquisition and attention. Some regions, specifically the dACC and the right DLFC, are involved in goal or task-directed behaviour, particularly task monitoring and error detection (Carter et al., 1998). Parietal regions, particularly the IPS, are typically associated with visual short-term memory (e.g., Xu and Chun, 2006) but also in the allocation of visuo-spatial attention (e.g., Connolly et al., 2016), as well as multisensory attentional processes (Macaluso et al., 2000; Anderson et al., 2010). However, regions within the IPS have also been implicated in the crossmodal transfer of information for determining visuo-motor action on objects (Sakata et al., 1997; Culham and Kanwisher, 2001) as well object properties (see e.g., Grefkes et al., 2002; Saito et al., 2003). Thus, it is feasible that in the current task, which involved visual categorisation of textures, the IPS may have been involved in controlling attention to information most relevant to the categorisation task which, as a consequence of training, may have been multisensory (i.e., from both the visual and tactile properties of the textures). In general, our finding of reduced activation in this region is consistent with models of learning that suggest the IPS is involved in cognitive or attentional control (Gilbert et al., 2001; Sigman et al., 2005). Processing in these prefrontal and parietal areas may therefore provide the neural ‘scaffolding’ required to facilitate learning and performance in a task (Kelly and Garavan, 2004).

Although not entirely consistent with some cognitive models of task proficiency (Chein and Schneider, 2012) we found reduced activation in lower-level regions of the brain, including large areas within the occipital lobe and fusiform gyrus following training. These activations are likely to be associated with repeated presentation of image-based or somatosensory properties of the texture materials themselves, as suggested by the so-called ‘sharpening model of response suppression’ (Wiggs and Martin, 1998; Grill-Spector et al., 2006). For example, Vuilleumier et al. (2002) reported reduced activation in early visual regions following repetition priming of images of both real and nonsense objects but found object-selective responses in anterior fusiform regions related to object meaning. In other words, whereas the reduced activation in occipital cortex may be related to stimulus repetition *per se*, the perceptual categorisation of objects resulted in activation in more anterior regions of the brain. This finding is consistent with our results which suggest that the learned perceptual representation of the texture categories may be associated with increases in activation within stimulus-specific regions of the brain but stimulus repetition may result in a general decrease in activation in occipital areas.

Functional cortical plasticity at the level of sensory processing has been identified predominantly in studies involving visual perceptual learning (Gibson, 1963; Ahissar and Hochstein, 1997; Carmel and Carrasco, 2008; Sasaki et al., 2010). Indeed, some fMRI studies have provided evidence that visual training can increase responses in primary visual cortex that are specific to trained but not untrained stimuli (e.g., Schwartz et al., 2002; Furmanski et al., 2004; Jehee et al., 2012). Other studies have also reported stimulus-specific activity in higher visual regions. For example, activity in the fusiform gyrus, which is implicated in face processing (Kanwisher et al., 1997), is reported to decrease in response to visually familiar but not unfamiliar faces (Dubois et al., 1999; Rossion et al., 2001, 2003; Kosaka et al., 2003; Gobbini and Haxby, 2006; see Natu and O’Toole, 2011 for a review). Furmanski et al. (2004) proposed several neurophysiological mechanisms which could mediate this process, such as an increase in the firing rate of neurons or the recruitment of additional neurons following training. However, the results of other studies suggest that the effect of visual training on activation in visual regions may generalise to untrained stimuli. For example, Schiltz et al. (1999) reported a decrease in functional activation (using positron emission tomography, PET) in several regions within the occipital cortex, including the fusiform gyrus, in response to both trained and untrained stimulus orientations (see also Schiltz et al., 2001 for a similar finding). It is not clear what is the basis for these inconsistent findings although differences in task demands or stimulus sets used for training are known to affect the learning process more generally (Green and Bavelier, 2008). It is also possible that the specific neurophysiological architecture of the visual cortex may mean that functional modifications with practise are distinct from those observed in other sensory or motor domains (Schiltz et al., 2001; Kelly and Garavan, 2004).

More pertinent to our own findings are studies involving multisensory perceptual learning. For example, Powers et al. (2012) observed decreases in activation in both visual and auditory cortices following training on an audiovisual simultaneity judgement task. Interestingly, they also reported increased functional connectivity between these regions and the superior temporal sulcus, implying the formation of a more efficient neural network in parallel with perceptual learning (see also Engel et al., 2012). In contrast, studies of multisensory expertise, for example musical training, have reported greater activation in specific sensory regions of the brain (Bangert and Altenmüller, 2003; Hasegawa et al., 2004; see also Herholz and Zatorre, 2012). Finally, crossmodal associations have also been reported in response to perceptual learning within a specific sensory domain, such as functional modulations within the visual ventral pathway following training on an auditory pattern recognition task (Poirier et al., 2006) or activation within the fusiform gyrus following training on Braille reading in sighted individuals (Debowska et al., 2016). These findings suggest that functional changes with perceptual training can be specific to the training modality itself, as well as the modality most dominant for that task (McGovern et al., 2016).

Similar to previous findings of an increase in activation in stimulus-specific regions of the brain, our results also suggest that crossmodal training was associated with an increase in activation within a particular region of the occipital lobe, namely the MOC, that was mainly lateralised to the left hemisphere. A subsequent analysis revealed that visual training was associated with unilateral, left activation of the medial occipital region whereas tactile training was associated with bilateral activation of this region. This apparent difference should be interpreted with some caution, however, as no interaction between categorisation task (pre- and post- training) and training modality was detected in the full model. Moreover, the lateralisation of these activations following practise on a texture categorisation task differ slightly from previous studies involving direct comparison of activation across visual and tactile conditions. For example, whereas Stilla and Sathian (2008) reported activation in the right MOC during haptic texture processing and bilateral activation during visual processing, in a later study Sathian et al. (2011) reported bilateral activation to tactile texture processing but activation in the left middle occipital gyri (MOG) during visual texture processing. Although both studies used similar fabric materials as stimuli for the texture tasks each study involved different perceptual tasks [i.e., shape versus texture task in the Stilla and Sathian study; localisation versus texture task in the Sathian et al. (2011) study] which may account for the functional differences. In a more direct comparison of crossmodal texture perception to our study, Eck et al. (2013) found an increase in activation in the left-posterior occipital cortex during bimodal compared to unimodal texture exploration but activation extended to the right hemisphere during tactile processing. Taken together with our own findings, these results suggest that familiarity with textures across both vision and touch is associated with greater activation within visual regions of the cortex, particularly MOC, but that the crossmodal processing of texture may affect the lateralisation of the activation. The finding that increases in activation are localised to a region within the occipital cortex, however, does suggest the intriguing possibility that these effects are related to perceptual processing *per se*, particularly texture perception, but that activations within these regions may be modulated by the specific requirements of the learned task.

Crossmodal training on a task involving texture categorisation, even when subsequently tested within one specific modality, can clearly affect processing in a wide network of regions. However, the extent to which these observed changes in activation is influenced by factors unrelated to sensory encoding or the task itself, such as handedness or stimulus discriminability, is not clear. Further research is required to elucidate the dynamic nature of these changes within sensory regions of the brain during and after intensive training, specifically on a perceptual task that typically involves multisensory processing, such as texture perception. Future studies may also help unravel the neural underpinnings of perceptual expertise in categorising stimuli from those associated with an increase in familiarity with the sensory aspects relevant to a particular task. In the meantime, our study provides some insight into the role of cognitive control as well as perceptual processes in the categorisation of naturalistic, multisensory textures with changes in brain function associated with both increased proficiency in the task as well as familiarity with the stimulus set.

## Ethics Statement

This study was carried out in accordance with the recommendations of the Good Research Practise guidelines issued by the Trinity College Research Committee, with written informed consent from all subjects in accordance with the Declaration of Helsinki. The protocol was approved by the School of Psychology Research Ethics Committee at Trinity College Dublin.

## Author Contributions

FN and CS-F designed the study. JS recruited participants and conducted the experiment. GO’C performed the data analyses. GO’C, AO’D, and FN wrote the manuscript.

## Conflict of Interest Statement

The authors declare that the research was conducted in the absence of any commercial or financial relationships that could be construed as a potential conflict of interest. The reviewer RA and handling Editor declared their shared affiliation.

## Footnotes

1 This pilot study (*N* = 16) also established that performance at categorising each of the fabrics as natural or synthetic (including 100% samples) was not at ceiling, either using vision or touch.

2 www.neurobs.com

3 Eigenvariate extraction in SPM12 from 5mm sphere around peak coordinates.
